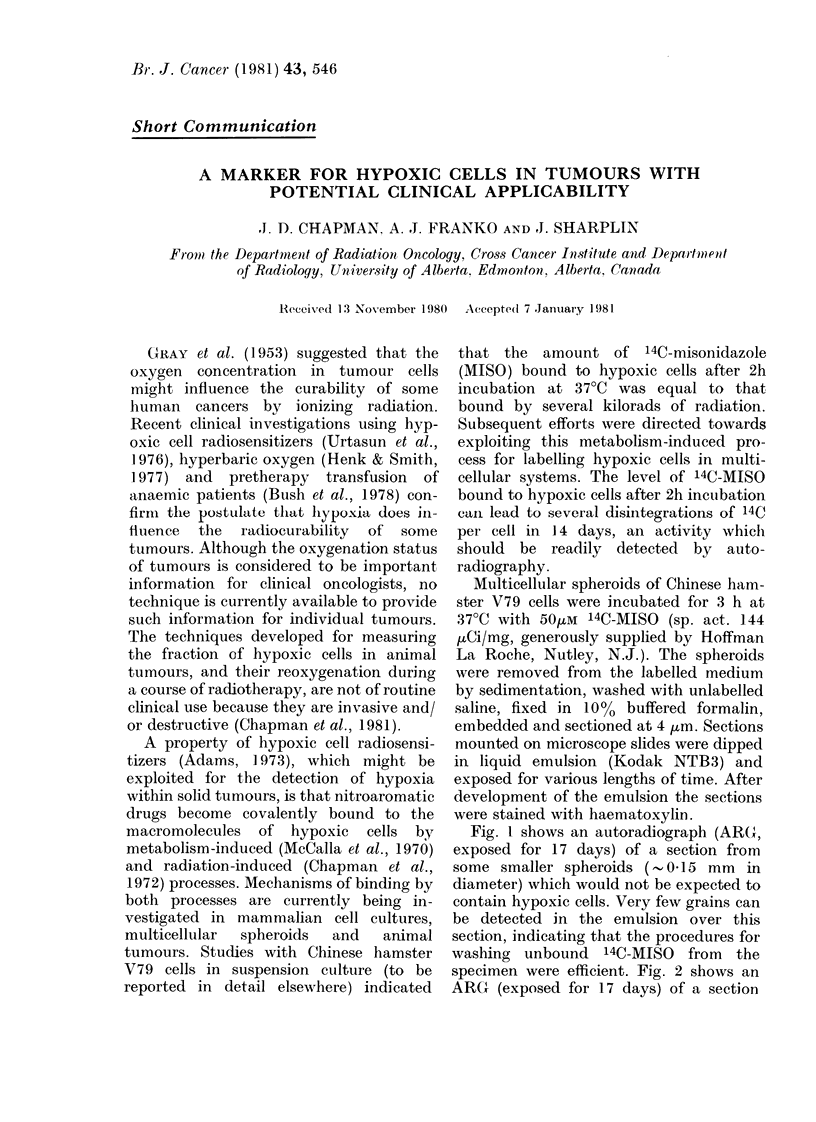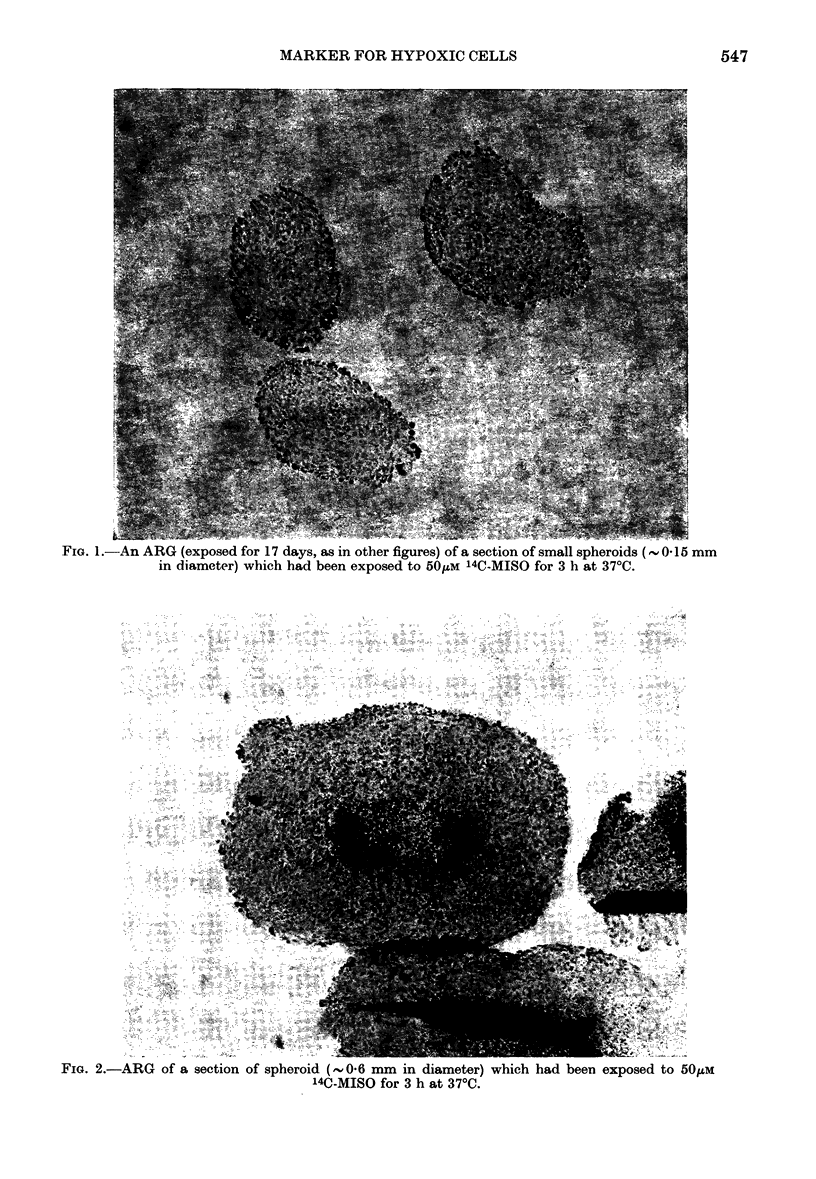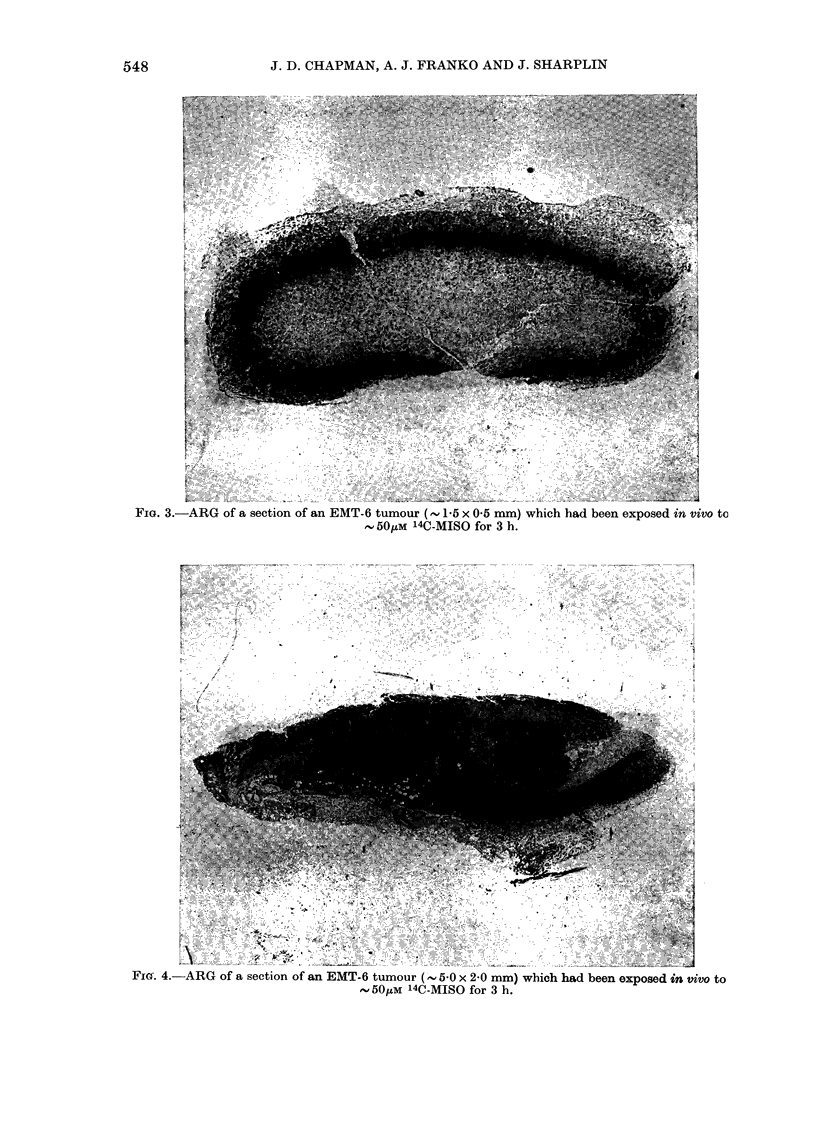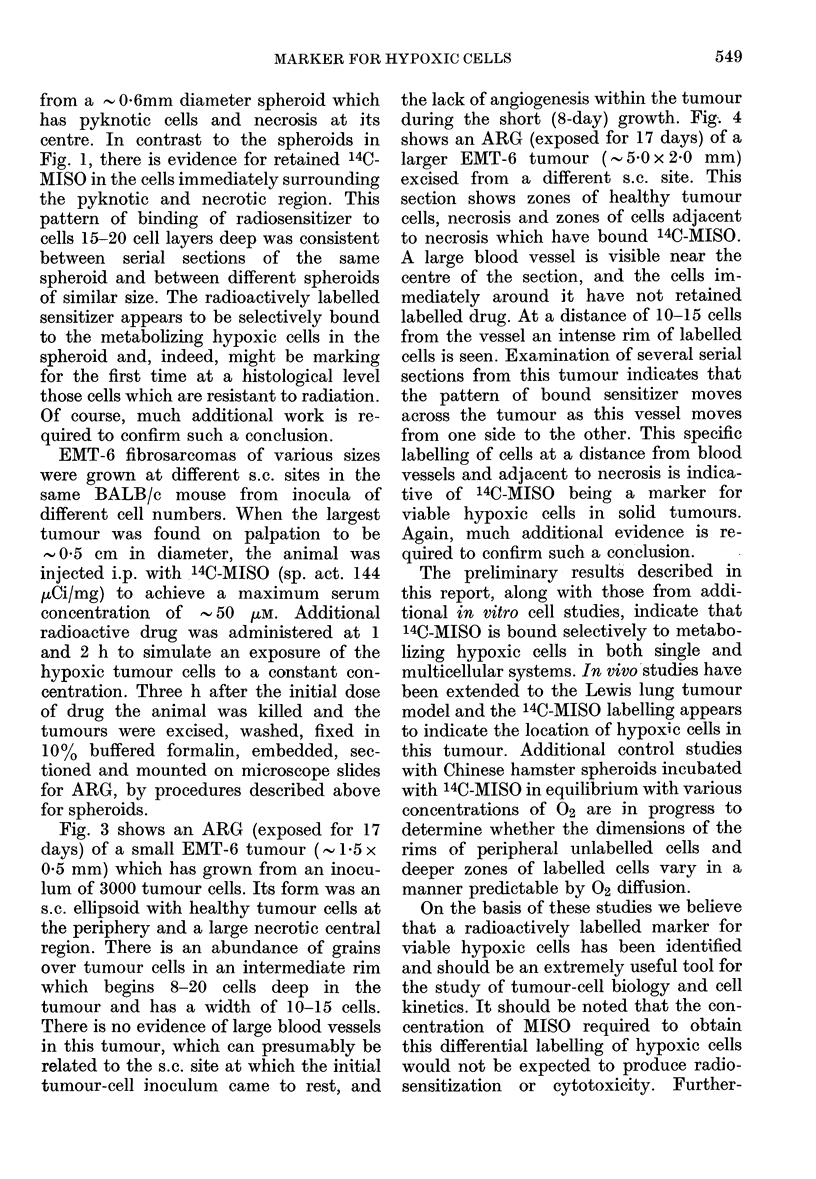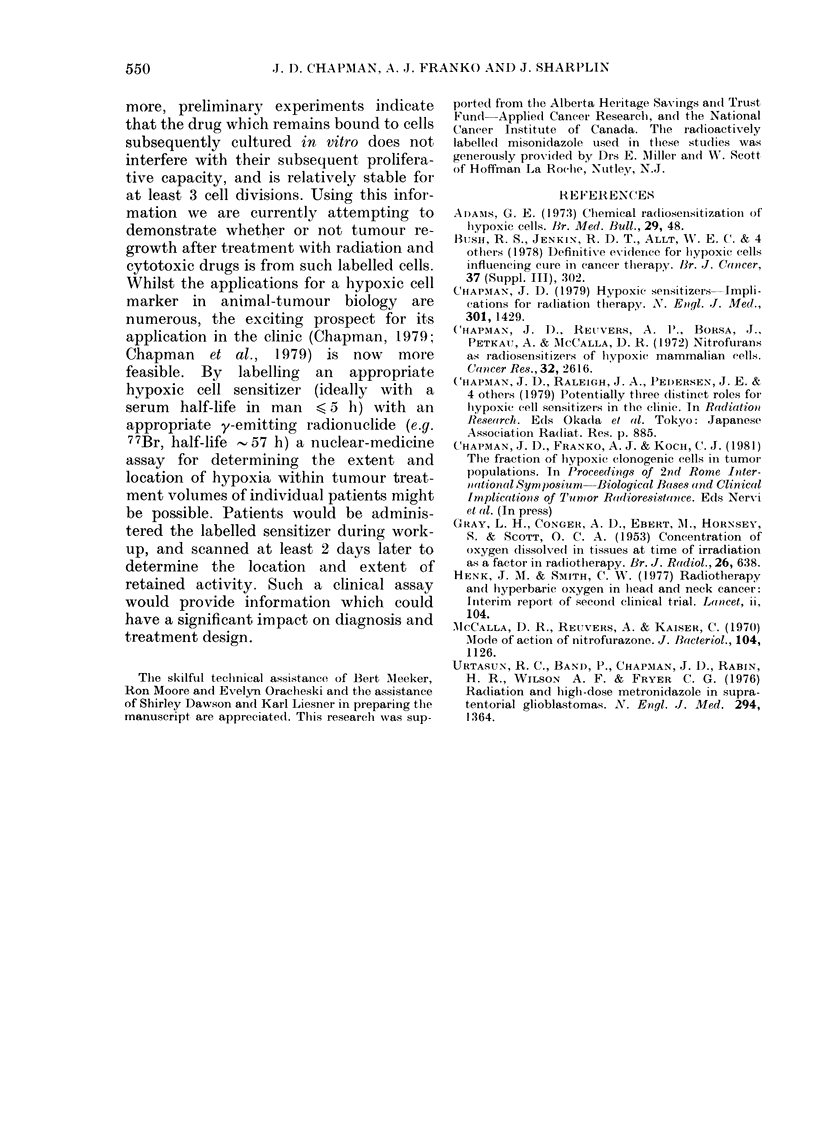# A marker for hypoxic cells in tumours with potential clinical applicability.

**DOI:** 10.1038/bjc.1981.79

**Published:** 1981-04

**Authors:** J. D. Chapman, A. J. Franko, J. Sharplin

## Abstract

**Images:**


*Br. J. Cancer* (1981) **43**, 546

***Short Communication***

### A MARKER FOR HYPOXIC CELLS IN TUMOURS WITH POTENTIAL CLINICAL APPLICABILITY

J. D. CHAPMAN, A. J. FRANKO AND J. SHARPLIN

*From the Department of Radiation Oncology, Cross Cancer Institute and Department of Radiology, University of Alberta, Edmonton, Alberta, Canada*

Received 13 November 1980 Accepted 7 January 1981

GRAY *et al.* (1953) suggested that the oxygen concentration in tumour cells might influence the curability of some human cancers by ionizing radiation. Recent clinical investigations using hypoxic cell radiosensitizers (Urtasun *et al.*, 1976), hyperbaric oxygen (Henk & Smith, 1977) and pretherapy transfusion of anaemic patients (Bush *et al.*, 1978) confirm the postulate that hypoxia does influence the radiocurability of some tumours. Although the oxygenation status of tumours is considered to be important information for clinical oncologists, no technique is currently available to provide such information for individual tumours. The techniques developed for measuring the fraction of hypoxic cells in animal tumours, and their reoxygenation during a course of radiotherapy, are not of routine clinical use because they are invasive and/or destructive (Chapman *et al.*, 1981).

A property of hypoxic cell radiosensitizers (Adams, 1973), which might be exploited for the detection of hypoxia within solid tumours, is that nitroaromatic drugs become covalently bound to the macromolecules of hypoxic cells by metabolism-induced (McCalla *et al.*, 1970) and radiation-induced (Chapman *et al.*, 1972) processes. Mechanisms of binding by both processes are currently being investigated in mammalian cell cultures, multicellular spheroids and animal tumours. Studies with Chinese hamster V79 cells in suspension culture (to be reported in detail elsewhere) indicated that the amount of $^{14}C$-misonidazole (MISO) bound to hypoxic cells after 2h incubation at 37°C was equal to that bound by several kilorads of radiation. Subsequent efforts were directed towards exploiting this metabolism-induced process for labelling hypoxic cells in multicellular systems. The level of $^{14}C$-MISO bound to hypoxic cells after 2h incubation can lead to several disintegrations of $^{14}C$ per cell in 14 days, an activity which should be readily detected by autoradiography.

Multicellular spheroids of Chinese hamster V79 cells were incubated for 3 h at 37°C with 50$\mu$M $^{14}C$-MISO (sp. act. 144 $\mu$Ci/mg, generously supplied by Hoffman La Roche, Nutley, N.J.). The spheroids were removed from the labelled medium by sedimentation, washed with unlabelled saline, fixed in 10% buffered formalin, embedded and sectioned at 4 $\mu$m. Sections mounted on microscope slides were dipped in liquid emulsion (Kodak NTB3) and exposed for various lengths of time. After development of the emulsion the sections were stained with haematoxylin.

Fig. 1 shows an autoradiograph (ARG, exposed for 17 days) of a section from some smaller spheroids ($\sim$0·15 mm in diameter) which would not be expected to contain hypoxic cells. Very few grains can be detected in the emulsion over this section, indicating that the procedures for washing unbound $^{14}C$-MISO from the specimen were efficient. Fig. 2 shows an ARG (exposed for 17 days) of a section

FIG. 1.—An ARG (exposed for 17 days, as in other figures) of a section of small spheroids ($\sim$0·15 mm in diameter) which had been exposed to 50$\mu$M $^{14}$C-MISO for 3 h at 37°C.

FIG. 2.—ARG of a section of spheroid ($\sim$0·6 mm in diameter) which had been exposed to 50$\mu$M $^{14}$C-MISO for 3 h at 37°C.

FIG. 3.—ARG of a section of an EMT-6 tumour (~1·5 × 0·5 mm) which had been exposed *in vivo* to ~50μM $^{14}$C-MISO for 3 h.

FIG. 4.—ARG of a section of an EMT-6 tumour (~5·0 × 2·0 mm) which had been exposed *in vivo* to ~50μM $^{14}$C-MISO for 3 h.

from a ~0·6mm diameter spheroid which has pyknotic cells and necrosis at its centre. In contrast to the spheroids in Fig. 1, there is evidence for retained $^{14}$C-MISO in the cells immediately surrounding the pyknotic and necrotic region. This pattern of binding of radiosensitizer to cells 15–20 cell layers deep was consistent between serial sections of the same spheroid and between different spheroids of similar size. The radioactively labelled sensitizer appears to be selectively bound to the metabolizing hypoxic cells in the spheroid and, indeed, might be marking for the first time at a histological level those cells which are resistant to radiation. Of course, much additional work is required to confirm such a conclusion.

EMT-6 fibrosarcomas of various sizes were grown at different s.c. sites in the same BALB/c mouse from inocula of different cell numbers. When the largest tumour was found on palpation to be ~0·5 cm in diameter, the animal was injected i.p. with $^{14}$C-MISO (sp. act. 144 $\mu$Ci/mg) to achieve a maximum serum concentration of ~50 $\mu$M. Additional radioactive drug was administered at 1 and 2 h to simulate an exposure of the hypoxic tumour cells to a constant concentration. Three h after the initial dose of drug the animal was killed and the tumours were excised, washed, fixed in 10% buffered formalin, embedded, sectioned and mounted on microscope slides for ARG, by procedures described above for spheroids.

Fig. 3 shows an ARG (exposed for 17 days) of a small EMT-6 tumour (~1·5 × 0·5 mm) which has grown from an inoculum of 3000 tumour cells. Its form was an s.c. ellipsoid with healthy tumour cells at the periphery and a large necrotic central region. There is an abundance of grains over tumour cells in an intermediate rim which begins 8–20 cells deep in the tumour and has a width of 10–15 cells. There is no evidence of large blood vessels in this tumour, which can presumably be related to the s.c. site at which the initial tumour-cell inoculum came to rest, and the lack of angiogenesis within the tumour during the short (8-day) growth. Fig. 4 shows an ARG (exposed for 17 days) of a larger EMT-6 tumour (~5·0 × 2·0 mm) excised from a different s.c. site. This section shows zones of healthy tumour cells, necrosis and zones of cells adjacent to necrosis which have bound $^{14}$C-MISO. A large blood vessel is visible near the centre of the section, and the cells immediately around it have not retained labelled drug. At a distance of 10–15 cells from the vessel an intense rim of labelled cells is seen. Examination of several serial sections from this tumour indicates that the pattern of bound sensitizer moves across the tumour as this vessel moves from one side to the other. This specific labelling of cells at a distance from blood vessels and adjacent to necrosis is indicative of $^{14}$C-MISO being a marker for viable hypoxic cells in solid tumours. Again, much additional evidence is required to confirm such a conclusion.

The preliminary results described in this report, along with those from additional *in vitro* cell studies, indicate that $^{14}$C-MISO is bound selectively to metabolizing hypoxic cells in both single and multicellular systems. *In vivo* studies have been extended to the Lewis lung tumour model and the $^{14}$C-MISO labelling appears to indicate the location of hypoxic cells in this tumour. Additional control studies with Chinese hamster spheroids incubated with $^{14}$C-MISO in equilibrium with various concentrations of $O_2$ are in progress to determine whether the dimensions of the rims of peripheral unlabelled cells and deeper zones of labelled cells vary in a manner predictable by $O_2$ diffusion.

On the basis of these studies we believe that a radioactively labelled marker for viable hypoxic cells has been identified and should be an extremely useful tool for the study of tumour-cell biology and cell kinetics. It should be noted that the concentration of MISO required to obtain this differential labelling of hypoxic cells would not be expected to produce radiosensitization or cytotoxicity. Furthermore, preliminary experiments indicate that the drug which remains bound to cells subsequently cultured *in vitro* does not interfere with their subsequent proliferative capacity, and is relatively stable for at least 3 cell divisions. Using this information we are currently attempting to demonstrate whether or not tumour regrowth after treatment with radiation and cytotoxic drugs is from such labelled cells. Whilst the applications for a hypoxic cell marker in animal-tumour biology are numerous, the exciting prospect for its application in the clinic (Chapman, 1979; Chapman *et al.*, 1979) is now more feasible. By labelling an appropriate hypoxic cell sensitizer (ideally with a serum half-life in man $\leqslant 5$ h) with an appropriate $\gamma$-emitting radionuclide (*e.g.* $^{77}$Br, half-life $\sim 57$ h) a nuclear-medicine assay for determining the extent and location of hypoxia within tumour treatment volumes of individual patients might be possible. Patients would be administered the labelled sensitizer during work-up, and scanned at least 2 days later to determine the location and extent of retained activity. Such a clinical assay would provide information which could have a significant impact on diagnosis and treatment design.

The skilful technical assistance of Bert Meeker, Ron Moore and Evelyn Oracheski and the assistance of Shirley Dawson and Karl Liesner in preparing the manuscript are appreciated. This research was supported from the Alberta Heritage Savings and Trust Fund—Applied Cancer Research, and the National Cancer Institute of Canada. The radioactively labelled misonidazole used in these studies was generously provided by Drs E. Miller and W. Scott of Hoffman La Roche, Nutley, N.J.

### REFERENCES

Adams, G. E. (1973) Chemical radiosensitization of hypoxic cells. *Br. Med. Bull.*, **29**, 48.

Bush, R. S., Jenkin, R. D. T., Allt, W. E. C. & 4 others (1978) Definitive evidence for hypoxic cells influencing cure in cancer therapy. *Br. J. Cancer*, **37** (Suppl. III), 302.

Chapman, J. D. (1979) Hypoxic sensitizers—Implications for radiation therapy. *N. Engl. J. Med.*, **301**, 1429.

Chapman, J. D., Reuvers, A. P., Borsa, J., Petkau, A. & McCalla, D. R. (1972) Nitrofurans as radiosensitizers of hypoxic mammalian cells. *Cancer Res.*, **32**, 2616.

Chapman, J. D., Raleigh, J. A., Pedersen, J. E. & 4 others (1979) Potentially three distinct roles for hypoxic cell sensitizers in the clinic. In *Radiation Research*. Eds Okada *et al.* Tokyo: Japanese Association Radiat. Res. p. 885.

Chapman, J. D., Franko, A. J. & Koch, C. J. (1981) The fraction of hypoxic clonogenic cells in tumor populations. In *Proceedings of 2nd Rome International Symposium—Biological Bases and Clinical Implications of Tumor Radioresistance*. Eds Nervi *et al.* (In press)

Gray, L. H., Conger, A. D., Ebert, M., Hornsey, S. & Scott, O. C. A. (1953) Concentration of oxygen dissolved in tissues at time of irradiation as a factor in radiotherapy. *Br. J. Radiol.*, **26**, 638.

Henk, J. M. & Smith, C. W. (1977) Radiotherapy and hyperbaric oxygen in head and neck cancer: Interim report of second clinical trial. *Lancet*, ii, **104.**

McCalla, D. R., Reuvers, A. & Kaiser, C. (1970) Mode of action of nitrofurazone. *J. Bacteriol.*, **104**, 1126.

Urtasun, R. C., Band, P., Chapman, J. D., Rabin, H. R., Wilson A. F. & Fryer C. G. (1976) Radiation and high-dose metronidazole in supratentorial glioblastomas. *N. Engl. J. Med.* **294**, 1364.